# Ultrasound-Assisted Extraction of Polysaccharides from *Volvariella volvacea*: Process Optimization and Structural Characterization

**DOI:** 10.3390/molecules23071706

**Published:** 2018-07-13

**Authors:** Feng-Jie Cui, Li-Sun Qian, Wen-Jing Sun, Jin-Song Zhang, Yan Yang, Na Li, Hai-Ning Zhuang, Di Wu

**Affiliations:** 1 School of Food and Biological Engineering, Jiangsu University, Zhenjiang 212013, China; 18352862482@163.com (L.-S.Q.); 18852862665@163.com (N.L.); 2 National Engineering Research Center of Edible Fungi, Shanghai Academy of Agricultural Sciences, Shanghai 201403, China; 18918162047@189.cn (J.-S.Z.); zhuanghaining@saas.sh.cn (H.-N.Z.); wudi@saas.sh.cn (D.W.); 3 Parchn Sodium Isovitamin C Co. Ltd., Dexing 334221, China

**Keywords:** *Volvariella volvacea*, polysaccharide, ultrasound-assisted extraction, optimization, response surface methodology, structural characterization

## Abstract

The aims of the present study were to optimize the operational parameters to maximize the yield of ultrasound-assisted polysaccharide extraction from *Volvariella volvacea* (straw mushroom) fruiting bodies by using for the first time one-factor-at-a-time and three-level Box-Behnken factorial designs. A maximum polysaccharide yield of 8.28 ± 0.23% was obtained under the optimized conditions of ultrasound power of 175 W, extraction temperature of 57 °C, extraction time of 33 min, and the ratio of liquid to raw material of 25:1, respectively. Compared to the hot-water extraction, the ultrasound-assistance favored the extraction of polysaccharides from *V. volvacea* for its higher polysaccharide yield and efficiency. Further preliminary polysaccharide structural characterization indicated that ultrasound treatment affected the monosaccharide compositions and ratios, and molecular weight range of polysaccharides extracted from *V. volvacea*.

## 1. Introduction

Edible/medicinal fungi or mushrooms, generally regarded as healthy foods and nutraceutical products, continue to attract growers’ and researchers’ attention, with an over 30-fold increase of world production and consumption ratios since 1978 [1,2,3]. Polysaccharides and polysaccharide-protein or peptide complexes are major health-benefitting macromolecules of edible/medicinal mushrooms, showing notable anti-tumor [4], immunomodulatory [5], anti-inflammatory [6] and antioxidative effects [7]. For their nontoxicity, natural origin and few side-effects, polysaccharides in mushrooms have been widely applied as nutraceuticals for nutraceutical product development or as ingredients for functional food production [8].

*Volvariella volvacea* (straw mushroom) is a typical tropical/subtropical species of edible mushroom presenting unique tasty/texture and nutritional values. With an estimated annual production of 330,000 tons China accounts for over 80% of the global production [9,10]. It contains 25.9–29.6% of protein and 2.24–3.6% of fat (per 100 g of dried fruiting bodies), and higher vitamin C levels than many vegetables and fruits [11]. Polysaccharides such as the (1→3)-β-d-glucans purified from cultured mycelia and fruiting body of *V. volvacea* have been proved to show potent antitumor activity against Sarcoma 180 solid tumors implanted in mice [12,13]. However, few references related to maximizing the *V. volvacea* polysaccharide extraction yield are available up to now.

Conventional methods to extract polysaccharides from mushrooms generally involve water or chemicals (acids or alkalis) along with high temperatures or pressures [14,15,16]. However, the long processing time (over 2 h) and low extraction yield and efficiency are the drawbacks for industrial application [17]. Hence, seeking an effective strategy to decrease the processing time and increase the extraction efficiency, and evaluating the polysaccharide structure changes occurring during extraction are still valuable objectives to exploit and apply these mushroom polysaccharides, both industrially and functionally.

Recently, enzyme-assistance, pulsed electric fields, ultrasound and microwave irradiation have been proposed for extracting maximum amounts of mushroom polysaccharides [18,19,20]. Among these, ultrasound-assisted extraction has received wide interest due to its easy operation, less energy input and improved efficiency under mild reaction conditions [21,22,23]. For example, ultrasound assistance (US) benefited the extraction of (1–3; 1–6)-β-d-glucan from *G. lucidum* fruiting bodies with a highest maximum yield of 107.13 mg within 1 h [24]. By comparing FTIR spectra and scanning electron microscopy results, it was proposed that ultrasound treatment destroyed the noncovalent intra- and inter-molecular bonds, and increased the polysaccharide dispersion or unfolding levels and hydrophilic groups became exposed to water molecules, which benefits the aqueous solubility of polysaccharide molecules [25]. Operational parameters including ultrasound power and frequency, extraction time and temperature would directly affect the polysaccharide extraction yields [26,27,28]. The maximum crude polysaccharide yield (9.66 ± 0.18%) of *Hibiscus rosa-sinensis* with strong in vitro scavenging activities on DPPH and hydroxyl radicals was obtained by optimizing the levels of factors such as ultrasonic power, extraction time, extraction temperature, and the water-to-raw material ratio by using response surface methodology (RSM) and Box–Behnken design (BBD) [29]. However, up to now, the references related to the extraction process optimization and characterization of *Volvariella volvacea* polysaccharide are still lacking. Hence, the present study mainly aimed to: (1) evaluate the application of an ultrasound-assisted extraction method to extract polysaccharides from *V. volvacea* fruiting bodies for the first time; (2) maximize the yield the *V. volvacea* polysaccharide by optimizing the process parameters using response surface methodology, and (3) initially characterize and compare the structural properties of polysaccharides extracted by conventional and ultrasound-assisted methods for further large-scale application.

## 2. Results and Discussion

### 2.1. One-Factor-At-A-Time Experiments

#### 2.1.1. Effect of Ultrasound Frequency

**Figure 1:**
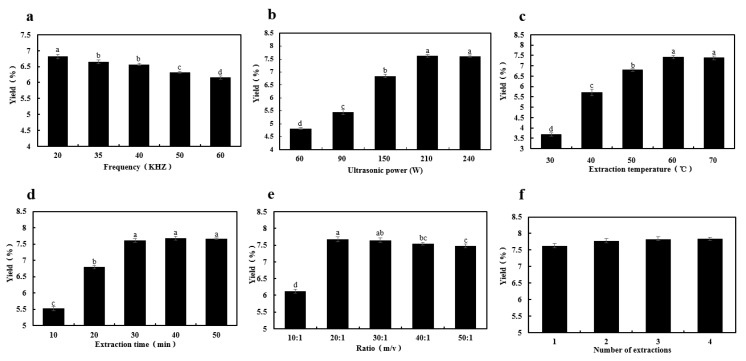
Effect of ultrasound frequency (**a**), ultrasound power (**b**), extraction temperature (**c**), extraction time (**d**), ratio of liquid to raw material (**e**) and number of extractions (**f**) on the yield of UAP-VVP (**a**–**d** represent the significant differences between various experiments for *p* < 0.05).

Ultrasound frequency affects the purity of extracted compounds and the process cost and efficiency [30]. The effect of ultrasound frequencies ranging from 20 KHz to 60 KHz on UAE-VVP yield was investigated with an ultrasound power of 150 W, extraction temperature of 50 °C, extraction time of 20 min, liquid/material ratio of 20:1, and number of extractions of 1. As shown in Figure 1a, an increase in the ultrasound frequency showed no significant influence on UAE-VVP yield, with a slight yield decrease from 6.82% to 6.16%. Similarly, an increase of ultrasonic frequency from 45 to 80 KHz decreased the yield of *Tricholoma matsutake* polysaccharides from 5.55% to 5.38% [31]. Therefore, an ultrasound frequency of 20 KHz was chosen for the further experiments.

#### 2.1.2. Effect of Ultrasound Power

Generally, ultrasound power could increase molecules’ solubility by destroying the intra-and inter-molecular bonds and increasing the contact efficiency between hydrophilic groups with extraction solvents [32]. Herein, five ultrasound powers ranging from 30 W to 240 W were used for investigating the influence of ultrasound power on the polysaccharide yield of *V. volvacea* fruiting bodies with the set conditions of ultrasound frequency of 20 KHz, extraction temperature of 50 °C, extraction time of 20 min, liquid/material ratio of 20:1, and number of extractions of 1, respectively. As shown in Figure 1b, the increase of ultrasound power from 60 W to 210 W improved the polysaccharide yield from 4.82% to 7.63%. However, further increase of ultrasound power to 240 W resulted in the slight decrease of polysaccharide yield to 7.59%. Similarly, ultrasound power of over 100 W had a negative effect on the extraction yield of *H. rosa-sinensis* leaves crude polysaccharide (HRLP) [29]. Therefore, around 150 W of ultrasound power was selected for further optimization with a Box-Behnken design.

#### 2.1.3. Effect of Extraction Temperature

Ultrasound extraction temperature affects the purity of extracted compounds and the process cost and efficiency [33]. Figure 1c presents the influence of temperature on the polysaccharide yield of *V. volvacea* fruiting bodies. The extraction was conducted at five extraction temperatures varying from 30 °C to 70 °C. *V. volvacea* polysaccharide yield was significantly affected by the temperature (*p* < 0.01). The maximum polysaccharide yield of 7.42% was obtained at 60 °C after 20 min of extraction. Generally, a higher temperature would benefit the polysaccharides solubility and extraction yield. For instance, Afshari et al. found that 95 °C was an optimal temperature for extraction of polysaccharides according for high yield and industrialization application [29]. Herein, around 50 °C of extraction temperature was selected for further statistical optimization.

#### 2.1.4. Effect of Extraction Time

Long extraction times usually benefit the extraction of polysaccharides from mushrooms or other raw materials. For example, okra curd polysaccharides’ (OCP) yield significantly increased to 15.7% after 6.5 h of extraction [34]. Figure 1d gives the effect of five extraction times (10, 20, 30, 40 and 50 min) on the yield of UAE-VVP with the ultrasound frequency of 20 KHz, ultrasound power of 150 W, extraction temperature of 50 °C, liquid/material ratio of 20:1, and number of extractions of 1. Polysaccharide yield increased rapidly from 5.52% to maximum level of 7.61% with the increase of extraction time from 10 min to 30 min. Further increases in extraction time (beyond 30 min) had no significant effect on the polysaccharide yield (*p* > 0.05). Similarly, with assistance of ultrasound, a time range of 10–30 min was suitable for *H. rosa-sinensis* leaves crude polysaccharide (HRLP) with a yield of approximately 8.8%, while an extraction time of 8 min resulted in the maximum *Boletus edulis* mycelial polysaccharides yield of 15.22% [29,35]. Therefore, an extraction time of around 30 min was selected for further optimization.

#### 2.1.5. Effect of Ratio of Water to Dried Fruiting Bodies

Different ratios (*v*/*w*) of liquid to dried fruiting bodies (10:1, 20:1, 30:1, 40:1, and 50:1) were used to investigate their effects on the polysaccharide yield. As shown in Figure 1e, the maximum polysaccharide yield was 7.68% when the ratio increased to 20:1. A further increase of ratio to 50:1 led to a slight decrease of polysaccharide yield. Generally, an increase of water to dried fruiting body would lead to an increase in the extraction working volume and energy input for evaporation, which is not economical. Hence, ratios of liquid to dried fruiting bodies from 10:1 to 30:1 were selected for further BBD optimization of polysaccharide extraction.

#### 2.1.6. Effect of Number of Extractions

As shown in Figure 1f, number of extractions was set from 1 to 4 times when other extraction conditions were fixed as ultrasound frequency of 20 KHZ, ultrasound power of 150 W, extraction time of 30 min, extraction temperature of 50 °C and liquid/material ratio of 20:1. The one time extraction yield of UAW-VVP was 7.63%. Further increasing the number of extractions to 4 seemed to have no significant effect, with the yield of polysaccharide increasing to only 7.84% (*p* > 0.05). Hence, extraction for one time was chosen for further experiments.

### 2.2. Optimization of the Parameters by RSM

Response surface methodology provides an empirical modeling for elucidating the effect of the independent variables, and interactive effects of each independent variable on the response [36]. In order to systemically understand the relationships between ultrasound power (X_1_), extraction temperature(X_2_), extraction time (X_3_) and ratio of liquid to dried fruiting bodies (X_4_) for UAE-VVP yield, a 3-level-4-factor Box-Behnken Design (BBD) was applied with 29 experimental runs (Table 1). Table 1 shows a considerable variation in the polysaccharide yields under different extraction conditions. The polysaccharide yield from *V. volvacea* fruiting bodies ranged from 5.13% to 8.21%, and the runs #12 and #27 had the maximum and minimum yield values, respectively. The coefficient of determination (R^2^) and adjusted coefficient of determination (R^2^a) were calculated as 98.81% and 97.61%, respectively. This indicated that, the accuracy and general predictive ability of the polynomial model was good, and analysis of the UAE-VVP yield trends using the model was considered to be reasonable [37].

According to the regression coefficients, along with the corresponding *p*-values, for the model of the extraction yield of *V. volvacea* polysaccharide, the linear coefficients (X_1_, X_2_, X_3_ and X_4_), quadratic coefficients (X_1_^2^, X_2_^2^, X_3_^2^, and X_4_^2^) and interaction coefficients(X_1_X_2_ and X_2_X_3_) were significant at the 1% level. The combined effect of the process variables was statistically significant (model *F*-value = 82.77, *p*-value < 0.0001).The second-order polynomial model for UAE-VVP yield(Y) was regressed by only considering the significant terms and was shown as below:

Y_UEA-VVP yield_ = 7.63 + 0.82X_1_ + 0.85X_2_ + 0.42X_3_ + 0.15X_4_ − 0.16X_1_X_2_ − 0.055X_2_X_3_ + 0.17X_2_X_4_ − 0.33X_1_^2^ − 0.44X_2_^2^ − 0.28X_3_^2^ − 0.42X_4_^2^ (1)

3D response surface plots and their corresponding contour plots were drawn on the basis of the model equation, to investigate the interactions among the variables and to determine the optimum concentration of each factor for maximum UAE-VVP yield. The contour plots affirm that the objective function is unimodal in nature, which shows an optimum in the boundaries.

**Figure 2:**
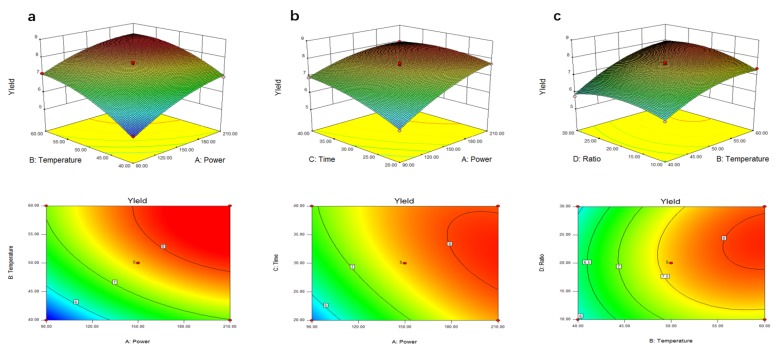
Response surface plots showing effects of ultrasound power (**a**), extraction temperature (**b**), extraction time and Ratio of liquid to raw material (**c**) on the yield of UAE-VVP.

Figure 2a presents the interaction relationship between the two independent variables (ultrasound power and extraction temperature) and the response variable (UAE-VVP yield) by keeping the other independent variables (extraction time and ratio) as constants (30 min and 20:1, respectively). It was obvious that the UAE-VVP yield showed the corresponding significant changes when ultrasound power and extraction temperature were subjected to small alterations. An increase in the UAE-VVP yield could be significantly achieved with the increases of ultrasound power and extraction temperature. Under certain condition (ultrasound power over 135 W, coded as −0.25~1; extraction temperature over 48 °C, coded as −0.20~1), a maximal contour (Y_UAE-VVP yield_ = 8.21%) could be determined (Figure 2a), meaning that further increases of ultrasound power and extraction temperature would not increase the polysaccharide yield levels significantly, which were in accordance with the results obtained in one-factor-at-a-time experiments.

Figure 2b describes the effects of X_1_ (ultrasound power) and X_3_ (extraction time) on yield level and indicated when X_1_ (ultrasound power) and X_3_ (extraction time) were in the ranges of 0.41~1 (coded value), and −0.50~0.90 (coded value), the yield was at a high level of 8.21%. The response curves demonstrated that the response value changed significantly with the low level of X_1_ (ultrasound power) and X_3_ (extraction time). In addition, the response curves were comparatively smooth when the X_1_(ultrasound power) and X_3_ (extraction time) were all in a high level.

Figure 2c shows the effects of X_2_ (extraction temperature) and X_4_ (liquid/material ratio) on polysaccharide extraction. When X_2_ (extraction temperature) and X_4_ (liquid/material ratio) ranged from 0.50 to 1 (coded value), and −0.20 to 0.80 (coded value), the yield increased to its relatively-highest value of 8.21%, consistent with the result seen in our one-factor-at-a-time experiments. Figure 2c also suggests that further increases over 55 °C and 28:1 would not increase the polysaccharide yield level significantly. Thus, in order to obtain the higher extraction yield, an appropriate level of X_2_ (extraction temperature) and X_4_ (liquid/material ratio) was essential. A maximum UAE-VVP yield of 8.32% by using ultrasound power (X_1_) of 172.13 W, extraction temperature (X_2_) of 56.68 °C, extraction time(X_3_) of 32.98 min, ratio of liquid to raw material (X_4_) of 24.45:1, respectively, could be predicted with the Design Expert software.

#### Verification of Predictive Model

The availability of the regression model (Equation (1)) of the UAE-VVP yield was tested using the calculated optimal culture composition, *viz.* ultrasound power 175 W, extraction temperature 57 °C, extraction time 33 min, ratio of liquid to raw material (X_4_) of 25:1, with triplicate experiments. The mean value of UAE-VVP yield was 8.28 ± 0.23%, which agreed well with the predicted value (8.32%). As a result, the models developed were considered to be accurate and reliable for predicting the UAE-VVP yield from *V. volvacea* fruiting bodies. Compared the polysaccharide (HWE-VVP) yield of 7.56% with 100 °C hot water for 2 h, the UAE method was attractive for its higher polysaccharide yield and efficiency in a shorter time (approximately 33 min). 

### 2.3. Comparison of the Structural Characteristics of Polysaccharides Extracted with Hot Water and Ultrasound-Assisted Methods

To find the possible structural changes of polysaccharides during the ultrasound treatment, the monosaccharide compositions and ratios, carbohydrate contents, average molecular weights and spectra of UAE-VVP were determined with those of HWE-VVP as a comparison. The *V. volvacea* polysaccharide fractions were obtained with hot water (HWE-VVP) and ultrasound-assisted (UAE-VVP) extractions followed with ethanol precipitation. The total carbohydrate contents in these polysaccharide fractions were determined as 65.82% and 60.78%, respectively. Table 2 showed monosaccharide compositions and ratios in UAE-VVP and HWE-VVP. Two polysaccharides contained the same monosaccharide compositions of d-glucose, d-mannose and d-galactose. d-Glucose is the main sugar, with ratios of 6.58 and 4.79 in UAE-VVP and HWE-VVP, respectively, which indicated that the main chain of these polysaccharides was possibly a glucan. The ultrasound treatment possibly broke the side chain containing d-mannose and d-galactose, which resulted in the increase of glucose ratio (6.58) in UAE-VVP. 

The average molecular weights of UAE-VVP and HWE-VVP were determined by HPSEC and shown in Table 3. UAE-VVP showed three fractions in the HPSEC chromatography with Mws and content percentages of 255.42 KDa, 2.69 KDa, 0.1167 KDa and 24.55%, 46.87%, 28.58%, respectively, while HWE-VVP had two peaks with Mws and content percentages of 357.56 KDa, 2.47 KDa, and 92.08%, 7.92%, respectively. The results also proved that the ultrasound treatment possibly partially destroyed the polysaccharide chains to produce a fraction in UAE-VVP. The lower extraction temperature for UAE also possibly contributed to the lower M_W_ of UAE-VVP, which was less effective for extraction and dissolution of the higher-M_W_ polysaccharides [38]. Similarly, the 120-min sonication decreased the molecular weight of waxy maize starch from around 3 × 10^6^ to 2 × 10^5^ [39]. Cheung et al. also found that all the polysaccharide–protein (PSP) complexes from three medicinal mushrooms (*Grifola frondosa*, *Coriolus versicolor* and *Lentinus edodes*) using ultrasound-assisted extraction (UAE) showed an increase of lower-M_W_ peaks and a decrease of higher-M_W_ peaks in peak area and number, or the shift of M_W_ distribution from high to low M_W_ range compared with those by how water extraction [40].

**Figure 3:**
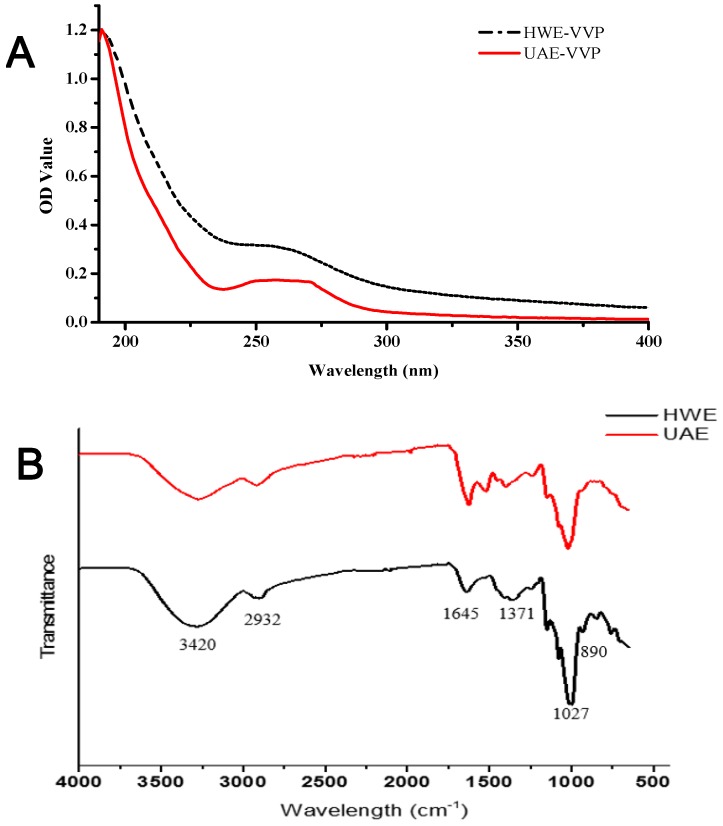
UV spectrograms (**A**) and Fourier transform infrared spectra (**B**) of HWE-VVP and UAE-VVP.

The UV spectra and FTIR-ATR spectra of UAE-VVP and HWE-VVP are shown in Figure 3. There was no UV absorption at the characteristic absorption peak of 260 nm and 280 nm, indicating that the sample contained extremely low content of proteins or nucleic acids (Figure 3A). Figure 3B gives the FTIR-ATR spectra of UAE-VVP and HWE-VVP. The large broad band at 3420 cm^−1^ corresponds to the typical hydroxyl group (O-H) absorption [41]. The intense band around 2932 cm^−1^ was attributed to the presence of the C-H stretching vibration [42]. The special characteristic of the absorption caused by asymmetric stretching vibration of carbonyl (C=O) in polysaccharides appeared at 1645 cm^−1^. The minor peaks at 1371 cm^−^^1^ and 1027 cm^−1^ confirmed the variable angle vibration of C-O-H. Otherwise, the minor peak noted at 890 cm^−1^ indicated the presence of the C-O-C stretching of glycosidic linkages [43]. The FT-IR characterization of UAE-VVP thus displayed the typical absorption peaks of polysaccharides [44]. There were no significant differences in the FTIR spectra of UAE-VVP and HWE-VVP. A similar phenomenon was also observed by Yan et al., whereby no considerable differences in the chemical structures of three *Corbicula fluminea* polysaccharides were observed after using ultrasound with three-phase partitioning (USTPP), ultrasound extraction (USE) and three-phase partitioning (TPP) [25].

## 3. Materials and Methods

### 3.1. Materials

*Volvariella volvacea* (straw mushroom) was cultivated at 32 ± 2 °C and 90% of relative humidity by Jiangnan Biotech Co., Ltd. (Zhenjiang, China). The fruiting body at egg stage was harvested after 4-week cultivation, freezing-dried to constant weight, and ground into powder (approximately 80-mesh size) with an electric mill for further extracting polysaccharides. Standard monosaccharides including glucose, xylose, arabinose, rhamnose, mannose, galactose and inositol were purchased from Sigma (St. Louis, MO, USA). Other chemicals and solvents were of analytical grade and used without further purification.

### 3.2. Polysaccharide Extraction

#### 3.2.1. Hot Water Extraction (HWE)

**Figure 4:**
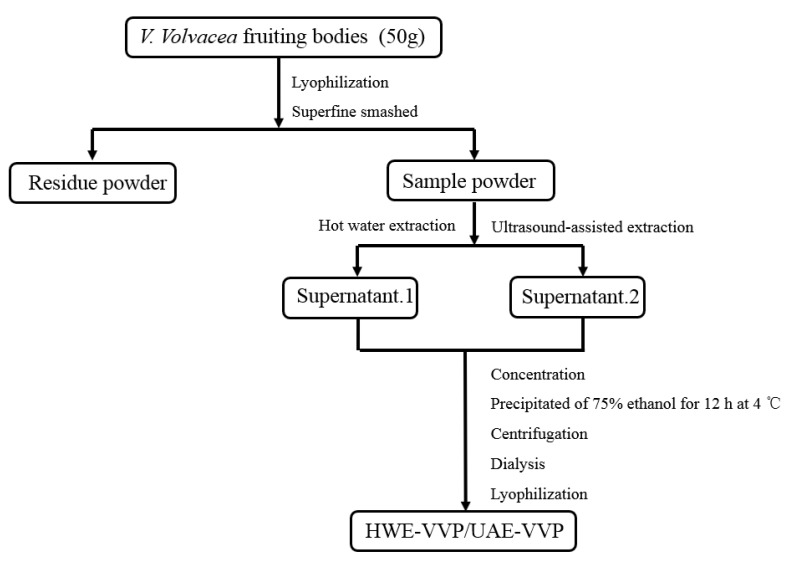
Stepwise scheme of *V. volvacea* polysaccharide extraction using ultrasound-assisted extraction (UAE) and hot water extraction (HWE).

As shown in Figure 4, hot water extraction (HWE) were carried out twice by mixing 50 g of ground *V. volvacea* fruiting bodies with 1000 mL of distilled water, heating for 100 °C for 2 h using a water bath, and centrifuging at 10,000× *g* for 20 min. The combined supernatants were concentrated under reduced pressure and precipitated with a final concentration of 75% ethanol for 12 h at 4 °C. The precipitation was collected by centrifugation (10,000× *g*, 15 min), followed by dialyzing against distilled water, and lyophilized to yield crude polysaccharides (HWE-VVP).

#### 3.2.2. Ultrasound-Assisted Extraction (UAE)

Ultrasound-assisted extraction (UAE) was conducted by using ultrasound equipment consisting of an ultrasonic reaction chamber (internal dimensions: 240 mm width × 207 mm length × 215 mm height, Shangjia Biotechnology Co., Wuxi, Jiangsu, China) equipped with three alternating dual-frequency plates, a water bath for balancing the treatment temperature, and a PLC control panel [45]. The ultrasound generators were installed at three sides of the bath reactor. The maximum output acoustic power of each plate is 300 W.

Fifty grams of ground *V. volvacea* fruiting bodies was mixed with distilled water and placed in reaction chamber for ultrasonic extraction. The other procedures to obtain the polysaccharide (UAE-VVP) with ultrasound-assisted extraction method were similar to those of HWE, including centrifugation to collect the supernatant, reduced pressure concentration, ethanol precipitation, dialysis against distilled water, and lyophilization.

### 3.3. Optimization of Extraction Parameters

One-factor-at-a-time experiments were conducted to evaluate the effects of ultrasound frequency (from 20 KHz to 60 KHz), ultrasound power (from 60 W to 240 W), extraction temperature (from 30 °C to 70 °C), extraction time (from 10 min to 50 min), number of extractions (from 1 to 4 times) and liquid/solid ratio (from 10:1 to 50:1) on the polysaccharide yields of *V. volvacea* fruiting bodies. 

Response surface methodology (RSM) was used to investigate the influence of ultrasound power (X_1_), extraction temperature (X_2_), extraction time (X_3_) and liquid/solid ratio (X_4_) on the polysaccharide yields of *V. volvacea* fruiting bodies. A Box-Behnken design with four factors and three levels was used for fitting a second order response surface [46]. 

Table 1 gave the factors, their values, and the experimental design, respectively. A mathematical model, describing the relationships between the response (the polysaccharide yield) and the process parameters in second order equation, was developed as follows:

(2) $$Y=A_{0}+\sum A_{i}X_{i}+\sum A_{ii}X_{i}^{2}+\sum A_{ij}X_{i}X_{j}$$

where *Y* is the predicted response variable; *A*_0_, *A_i_*, *A_ii_*, *A_ij_* are constant regression coefficients of the model, and *X_i_*, *X_j_* (*i* = 1, 4; *j* = 1, 4, *i* ≠ *j*) represent the independent variables (extraction parameters) in the form of coded values. The accuracy and general ability of the above polynomial model could be evaluated by the coefficient of determination R^2^.

### 3.4. Analysis

#### 3.4.1. Polysaccharide Yield

Polysaccharide yield (%) of *V. volvacea* fruiting bodies was calculated by dividing the amount of extracted crude polysaccharide by the weight of *V. volvacea* fruiting bodies. 

#### 3.4.2. Carbohydrate Content and Monosaccharide Composition Analysis

Carbohydrate content of HWE-VVP or UAE-VVP was determined at 490 nm by the phenol-sulfuric acid method with D-glucose as a standard [47]. Monosaccharide compositions and their ratios in HWE-VVP or UAE-VVP were determined by absolute hydrolysis [48]. Briefly, 30 mg HWE-VVP or UAE-VVP were hydrolyzed with 0.3 mL 72% (*w*/*v*) sulfuric acid for 30 min at 30 °C, followed with addition of 8.4 mL of distilled water and incubation at 121 °C for 1 h. The hydrolysate was neutralized with CaCO_3_, evaporated to dryness, and acetylated with Ac_2_O-pyridine at 90 °C for 30 min. Xylose, glucose, arabinose, rhamnose, mannose, and galactose were derivatized as standard. Inositol was used as an internal standard. The resulting alditol acetate was analyzed by gas chromatography using an Agilent Technologies 7890A Network gas chromatograph using a capillary column (HP-1 2.1 m × 0.2 mm, i.d.) and a flame ionization detector (FID) (Hewlett-Packard, Avondale, PA, USA) with nitrogen as carrier gas (50 mL/min). The temperatures for injector and the detector were kept at 280 °C and 300 °C, respectively. The products were identified by typical retention times of corresponding standards. 

#### 3.4.3. Molecular Weight Analysis by Gel Permeation Chromatography

The molecular weight of HWE-VVP or UAE-VVP was determined by high performance size exclusion chromatography (HPSEC) on a Waters 1525 HPLC system (Waters Corporation, Milford, MA, USA) equipped with an Ultrahydrogel Linear (7.8 mm × 300 mm, Waters Corporation) and a 2414 refractive index detector. HWE-VVP or UAE-VVP (2.0 mg) were dissolved by 1.0 mL NaNO_3_ (0.1 M) and loaded into the chromatography system (15 μL). The columns were maintained at 45 °C and eluted with 0.1 M NaNO_3_ at a flow rate of 0.9 mL/min. Preliminary calibration of the column was conducted by using standards dextrans (Sigma-Aldrich, St. Louis, MO, USA) with different molecular weights. The molecular weight (Mw) of HWE-VVP or UAE-VVP was calculated by comparison with the calibration curve [49].

#### 3.4.4. UV and FTIR

The aqueous solution of HWE-VVP or UAE-VVP was formulated into a 1 mg/mL aqueous solution and scanned with a UV spectrophotometer at a wavelength of 190–400 nm. The UV spectrum of the HWE-VVP or UAE-VVP solution was obtained at room temperature compared with water.

The Fourier Transform-Infrared Attenuated Total Reflectance (FTIR-ATR) spectra of HWE-VVP or UAE-VVP was detected using a Card-Fourier Infrared Spectrometer (Nicolet, Madison, WI, USA) to determine the chemical bonds, chemical nature and functional groups of extracted polysaccharides. The spectrum was recorded from 400 to 4000 cm^−1^ by accumulation of16 scans using resolution setting of 4 cm^−1^ and plotted as % transmittance *vs.* wavenumber [50]. 

### 3.5. Statistics

Each experiment was repeated three times using duplicate samples. The results were expressed as means ± standard deviations. Statistical comparisons were made by one-way analysis of variance (ANOVA), followed by Duncan’s multiple-comparison test [51,52]. Differences were considered significant when the *p*-values were <0.05 [53]. The 3D response surface and contour plot analysis were made by keeping one independent variable at constant level, changing the other two independent variables with Design Expert software version 11 (Stat-Ease, Inc., Minneapolis, MN, USA).

## 4. Conclusions

The present study presented the positive effects of an ultrasound-assisted method on the yield and efficiency of polysaccharide extraction from *Volvariella volvacea* fruiting bodies. The operational parameters and levels for *V. volvacea* polysaccharide extraction were selected and optimized by using one-factor-at-a-time and response surface methodology. Under optimal conditions of ultrasound power of 175 W; extraction temperature of 57 °C; extraction time of 33 min; and ratio of liquid to raw material of 25:1, respectively, the verified experimental value of polysaccharide yield was 8.28 ± 0.23%. UAE-VVP showed significant differences of monosaccharide compositions and ratios, and molecular weight range from those observed in HWE-VVP. Studies on the fine structure information and immunomodulating activities of UAE-VVP and HWE-VVP are ongoing in our lab for further evaluating the application of ultrasound-assisted method for polysaccharide extraction from *V. volvacea* fruiting bodies as potential functional ingredients in the health food and nutrition industries.

## Figures and Tables

**Table 1:** Coded and levels of independent variables and the response used in Box-Behnken design matrix.

Run	Ultrasound Power (W, X_1_)	Extraction Temperature (Degrees C, X_2_)	Extraction Time (min, X_3_)	Extraction Ratio (mL/g, X_4_)	Yield (%)
1	90(−1)	50(0)	30(0)	30(1)	6.08 ± 0.23
2	90(−1)	60(1)	30(0)	20(0)	7.10 ± 0.31
3	210(1)	50(0)	40(1)	20(0)	7.95 ± 0.18
4	150(0)	50(0)	30(0)	20(0)	7.54 ± 0.30
5	150(0)	40(−1)	40(1)	20(0)	6.55 ± 0.28
6	90(−1)	50(0)	20(−1)	20(0)	5.42 ± 0.36
7	150(0)	50(0)	30(0)	20(0)	7.51 ± 0.30
8	150(0)	50(0)	30(0)	20(0)	7.65 ± 0.24
9	150(0)	50(0)	20(−1)	30(1)	6.64 ± 0.28
10	90(−1)	50(0)	40(1)	20(0)	6.90 ± 0.23
11	90(−1)	50(0)	30(0)	10(−1)	5.97 ± 0.33
12	210(1)	60(1)	30(0)	20(0)	8.21 ± 0.35
13	210(1)	40(−1)	30(0)	20(0)	6.89 ± 0.38
14	150(0)	50(0)	30(0)	20(0)	7.75 ± 0.27
15	210(1)	50(0)	30(0)	30(1)	7.96 ± 0.19
16	150(0)	40(−1)	20(−1)	20(0)	5.74 ± 0.27
17	150(0)	60(1)	30(0)	30(1)	7.94 ± 0.33
18	150(0)	40(−1)	30(0)	10(−1)	5.88 ± 0.21
19	150(0)	50(0)	40(1)	10(−1)	7.08 ± 0.26
20	150(0)	50(0)	40(1)	30(1)	7.68 ± 0.31
21	150(0)	50(0)	30(0)	20(0)	7.68 ± 0.24
22	150(0)	60(1)	40(1)	20(0)	8.07 ± 0.28
23	150(0)	60(1)	30(0)	10(−1)	7.38 ± 0.37
24	150(0)	40(−1)	30(0)	30(1)	5.75 ± 0.21
25	210(1)	50(0)	30(0)	10(−1)	7.70 ± 0.24
26	210(1)	50(0)	20(−1)	20(0)	7.69 ± 0.32
27	90(−1)	40(−1)	30(0)	20(0)	5.13 ± 0.27
28	150(0)	60(1)	20(−1)	20(0)	7.48 ± 0.19
29	150(0)	50(0)	20(−1)	10(−1)	6.22 ± 0.24

**Table 2:** Monosaccharide composition of VVP extracted with ultrasound-assisted extraction (UAE) and hot water extraction (HWE).

	Molar Ratio		
	d-Mannose (22.008 min)	d-Glucose (22.898 min)	d-Galactose (23.545 min)
UAE-VVP	1	6.58	1.12
HWE-VVP	1	4.79	1.14

**Table 3:** Retention time, Mw and amount of soluble polysaccharide fractions extracted with ultrasound-assisted extraction (UAE) and hot water extraction (HWE).

Samples	Peak No	Retention Time (min)	M_W_ (KDa)	Area Percentages (%)
HWE-VVP	1	13.41	357.56	92.08
	2	16.98	2.47	7.92
UAE-VVP	1	13.17	255.42	24.55
	2	16.95	2.69	46.87
	3	19.37	0.12	28.58
